# T170. THE SUPERIOR LONGITUDINAL FASCICULUS: CAN CSD BASED TRACT DELINEATION AND NODAL ANALYSIS CLARIFY THE PRESENCE OF TARGETED DIVERGENT DEVELOPMENTAL STRUCTURAL CONNECTIVITY IN ADOLESCENTS REPORTING PSYCHOTIC EXPERIENCES

**DOI:** 10.1093/schbul/sby016.446

**Published:** 2018-04-01

**Authors:** Erik O’Hanlon, Niamh Dooley, Colm Healy, Mary Cannon

**Affiliations:** Royal College of Surgeons in Irel

## Abstract

**Background:**

Superior Longitudinal Fasciculus (SLF) differences are consistently reported in psychotic disorders. The SLF is a complex large bundle of association white matter fibers that bidirectionally connect caudal, temporal cortex and inferior parietal cortex to locations in the frontal lobe. Advances in tractography methodologies detail four discrete subdivisions (SLF I, II, III and IV, more often referred to as the arcuate fasciculus (AF)). Greater specificity of the SLF subdivisions and associated cognitive networks may clarify the mechanisms of divergent tract developmental and functional aspects associated with psychotic experience symptomology in population based samples of adolescents within the extended psychosis continuum

**Methods:**

A case-control sample of 25 adolescents reporting psychotic experiences versus 25 controls (mean age 13.7 years). We employed High Angular Resolution Diffusion Imaging (HARDI) based data with constrained spherical deconvolution (CSD) based fibre tractography to delineate the discrete subdivisions of the SLF including the arcuate fasciculous. Following tract identification, standard diffusion metrics, (fractional anisotrophy (FA), and Diffusivity measures MD, AD and RD), were assessed. A secondary supportive “along-tract” analysis to ascertain more subtle patterns of variation of tract integrity over the tract length was applied. White matter nodal analysis exploring the structural connectivity was applied to investigate additional functional networks recruited by and interconnected via the SFL. We investigate the ability of tractography of the SLF subdivisions and nodal analysis to identify possible differences between adolescents experiencing subclinical psychotic like experiences and those who don’t.

**Results:**

Our results agree with recent studies of the SLF I and AF (Fernandes-Miranda et al 2015) revealing a pattern of asymmetry of these tracts with more extensive tract bundles being consistently identified in the left hemisphere compared to the right. Along-tract analysis revealed subtle patterns of change in discrete subdivisions of the SLF while nodal analysis shows promise in its ability to define precise organisational networks

**Discussion:**

Delineating the SLF subdivisions may clarify potential developmental trajectories between frontal and parieto-temporal speech-related areas contributing to the pathogenesis of psychotic like experiences. These results reveal the presence of aberrant structural connectivity in young adolescents with psychotic experiences.

